# Supplementary material for: Use of a human small airway epithelial cell line to study the interactions of Aspergillus fumigatus with pulmonary epithelial cells
Source: mSphere. 2023 Aug 14;8(5):e00314-23. doi: 10.1128/msphere.00314-23 (PMC10597448; doi:10.1128/msphere.00314-23)
Supplement: Fig. S1 and 2 — Adherence of A. fumigatus to A549 and HSAE cells. [file msphere.00314-23-s0001.docx]

**
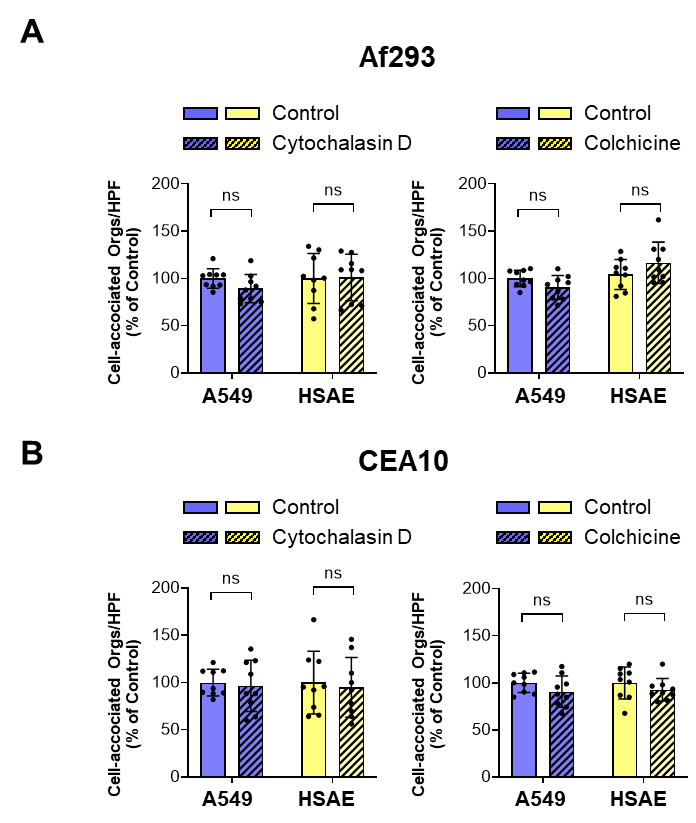
**

**Fig S1. Adherence *A. fumigatus* to A549 and HSAE cells is not affected by cytochalasin D and colchicine.**

(A and B) Effects of 0.6 μM Cytochalasin D and 0.5 μM colchicine on the cell-association (a measure of adherence) of *A. fumigatus* Af293 (A) and CEA10 (B) with A549 and HSAE cells.

Results are mean ± SD of 3 independent experiments, each performed in triplicate. orgs/HPF, organisms per high-powered field; ns, not significant by the unpaired Students t-test.

**
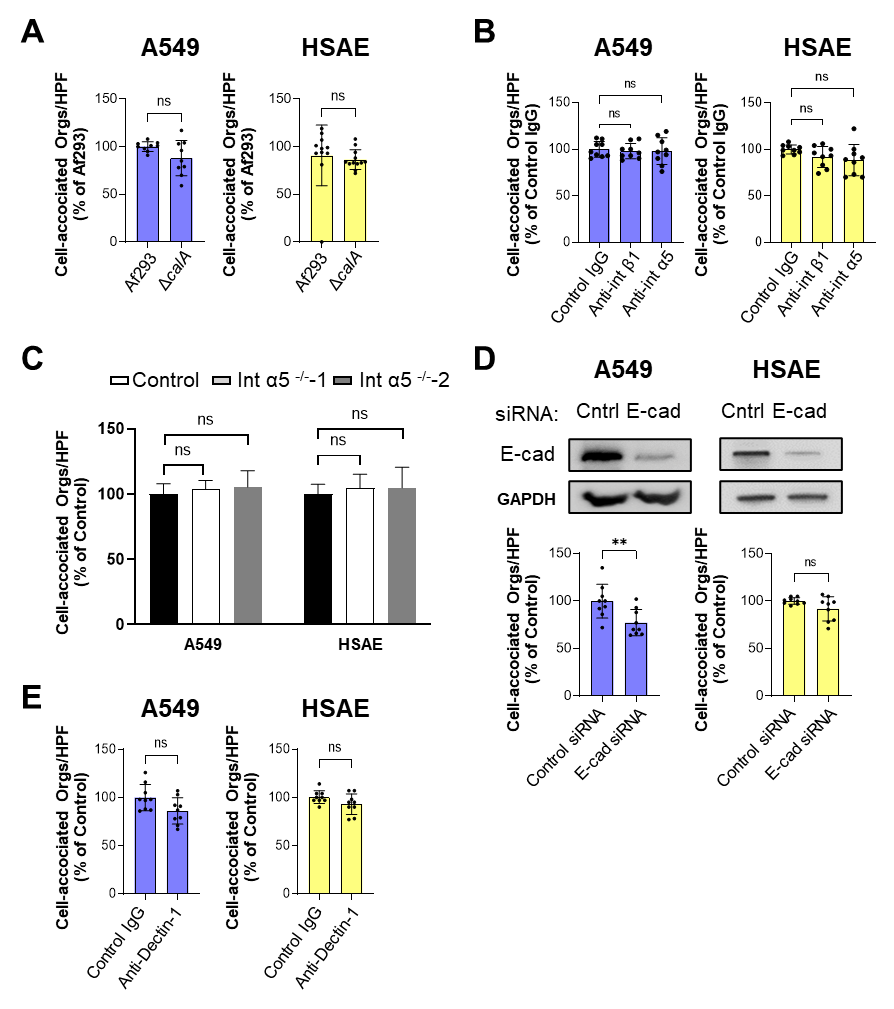
**

**Fig S2. Adherence of *A. fumigatus* to A549 and HSAE cells.**

(A) Adherence of the indicated *A. fumigatus* strains to A549 and HSAE cells.

(B) Effects of anti-β1 integrin and anti-α5 integrin antibodies on the adherence of *A. fumigatus* Af293 to A549 and HSAE cells.

(C) Deletion of integrin α5 has no effect on the adherence of *A. fumigatus* Af293 to A549 and HSAE cells.

(D) Effects of siRNA knockdown of E-cadherin on the adherence of *A. fumigatus* Af293 to A549 and HSAE cells. Top panels contain representative immunoblots of whole cell lysates showing siRNA knockdown of E-cadherin in A549 and HSAE cells.

(E) Effects of anti-Dectin-1 antibody on the adherence of *A. fumigatus* Af293 to A549 and HSAE cells.

Results are mean ± SD of 3 independent experiments, each performed in triplicate. orgs/HPF, organisms per high-powered field; ns, not significant; ***P* < 0.01 by unpaired Students t-test (A, D and E) and ANOVA with Dunnett’s test for multiple comparison (B and C).
