# Supplementary material for: Use of a human small airway epithelial cell line to study the interactions of Aspergillus fumigatus with pulmonary epithelial cells
Source: mSphere. 2023 Aug 14;8(5):e00314-23. doi: 10.1128/msphere.00314-23 (PMC10597448; doi:10.1128/msphere.00314-23)
Supplement: Table S1 — Summary of Aspergillus-host interactions. [file msphere.00314-23-s0002.docx]

| Cell line |  | A549 | | HSAE | |
| --- | --- | --- | --- | --- | --- |
| *Aspergillus* strain |  | Af293 | CEA10 | Af293 | CEA10 |
| Endocytosis | Live conidia | + | + | ++++ | ++++ |
|  | Live germlings | ++++ | ++ | ++ | ++ |
|  | Thi killed germlings | +++ | ++ | +/- | +/- |
|  | Cytochalasin D inhibition | ↓↓↓ | ↓↓ | ↓ | ↓ |
|  | Colchicine inhibition | ↓↓ | ↓↓ | ↓↓↓↓ | ↓↓↓↓ |
| Cell damage | Live organisms | ++ | ++ | ++++ | ++++ |
|  | Thi killed germlings | +/- | - | ++ | + |
|  | Para killed germlings | - | - | +++ | ++ |
|  | Culture filtrates | + | - | ++++ | + |
| Cytokine or chemokine expression | CXCL8 | ↑+++ | ↑+++ | ↑++ | ↑++ |
|  | CXCL1 | ↔++ | ↔++ | ↑+++ | ↑+++ |
|  | IL-6 | ↑+ | ↑+ | ↑+ | ↑+ |
|  | IL-1α | ↑++ | ↑++ | ↑++ | ↑++ |
|  | IL-1β | ↑++ | ↑++ | ↑++ | ↑++ |
|  | TNFα | ↑+ | ↑+ | - | - |
|  | GM-CSF | ↑++ | ↑++ | - | - |
|  | CCL2/MCP-1 | ↔+++ | ↔+++ | - | - |

Table S1. Summary of host cell endocytosis, damage, and cytokines or chemokines expression during *A. fumigatus* infection.

+ Indicates the different efficiency of endocytosis and damage or levels of cytokine or chemokine expression; ↓ Indicates the effects of the inhibitors; ↑ Indicates up-regulation; ↔ indicates no change; Thi, thimerosal; Para, paraformaldehyde.
