# Supplementary material for: Use of a human small airway epithelial cell line to study the interactions of Aspergillus fumigatus with pulmonary epithelial cells
Source: mSphere. 2023 Aug 14;8(5):e00314-23. doi: 10.1128/msphere.00314-23 (PMC10597448; doi:10.1128/msphere.00314-23)
Supplement: Table S2 — A. fumigatus strains used in this study. [file msphere.00314-23-s0003.docx]

| **Strain Name** | **Background** | **Genotype** | **Source** |
| --- | --- | --- | --- |
| Af293 | Wild-type | Wild-type | P. Magee, University of Minnesota |
| CEA10 | Wild-type | Wild-type | N. Keller, University of Wisconsin-Madison |
| Af293-*GFP* | Af293 | P*_gpdA_-GFP-ble* | Liu and Lee et al., 2016 |
| CEA10*-GFP* | CEA10 | P*_gpdA_-GFP-ble* | Liu and Lee et al., 2016 |
| Δ*pacC* | Af293 | Δ*pacC::hph* | Present study |
| Δ*pacC-Comp* | Af293 | Δ*pacC::hph+pacC-ble* | Present study |
| Δ*pacC-GFP* | Δ*pacC*-Af293 | P*_gpdA_-GFP-ble* | Present study |
| Δ*pacC-Comp-GFP* | Δ*pacC-Comp*-Af293 | P*_gpdA_-GFP-ptrA* | Present study |

Table S2. *A. fumigatus* strains used in this study.
