# Supplementary material for: Use of a human small airway epithelial cell line to study the interactions of Aspergillus fumigatus with pulmonary epithelial cells
Source: mSphere. 2023 Aug 14;8(5):e00314-23. doi: 10.1128/msphere.00314-23 (PMC10597448; doi:10.1128/msphere.00314-23)
Supplement: Table S3 — Primers used in this study. [file msphere.00314-23-s0004.docx]

Table S3. Oligonucleotides primers used in this study.

| Primer name | Primer sequence (5' - 3') |
| --- | --- |
| sgRNA-F | GCGTAAGCTCCCTAATTGGC |
| sgRNA-R | GAGCCAAGAGCGGATTCCTC |
| Cas9-F | CGAGACAGCAGAATCACCGC |
| Cas9-R | GTATTGGGATGAATTTTGTATGCAC |
| sgRNA-PacC-F1 | AGTAAGCTCGTCAGAATGAAGGGAGGATATCAGTTTTAGAGCTAGAAATAGCAAGT |
| sgRNA-PacC-R1 | TGATATCCTCCCTTCATTCTGACGAGCTTACTCGTTTCGTCCTCACGG |
| sgRNA-PacC-F2 | AGTAAGCTCGTCTGACAAGGCTGGATCGACATGTTTTAGAGCTAGAAATAGCAAGT |
| sgRNA-PacC-R2 | ATGTCGATCCAGCCTTGTCAGACGAGCTTACTCGTTTCGTCCTCACGG |
| Hyg-F | GGAAACACGATTCATCACAGTTCACTTGCCTTGATATCCTCCCTTCATTCTTGACCAAGAATCTATTGCATC |
| Hyg-R | CACCCGGTCAACTCCGTAGAAAGGGAAATGACAAGGCTGGATCGACATTGAGTGTGCTGGAATTCGCCCTTC |
| PacC-Screen-Up | TGTGACTTGCCTACCCTAAG |
| Hyg-Screen-R | AATAGGTCAGGCTCTCGCTG |
| PacC-Screen-F | ACATCACGTCTCACATCC |
| PacC-Screen-R | ATGGTATCCTGCATCTGC |
| PacC-Com-F | TCAGCTGCGGCCGCAAGGTACCTGATGTCGACGAGGTG |
| PacC-Com-R | ATGTCAGCGGCCGCTCGGTAGGTGAGTAGCAGGATCAG |
| Cas9-ScreenF | ATGGACAAGAAGTATAGCATCG |
| Cas9-ScreenR | GGAGTCAGACCAAGTGACAAC |
| sgRNA-ScreenF | TTGGCCCATCCGGCATCTGTA |
| sgRNA-ScreenR | CTCTGCTAAGCTATTCTTCTGC |
| ITGA5-sgRNA1-F | CACCGAATTCGGGTGAAGTTATCTG |
| ITGA5-sgRNA1-R | AAACCAGATAACTTCACCCGAATTC |
| ITGA5-sgRNA2-F | CACCGCACTAGCGGACACGATGGGG |
| ITGA5-sgRNA2-R | AAACCCCCATCGTGTCCGCTAGTGC |
| TEF1-F | GTGACTCCAAGAACGATCC |
| TEF1-R | AGAACTTGCAAGCAATGTGG |
| PacC-Realtime-F | TGACCAGGCTATGCTGCTG |
| PacC-Realtime-R | TGGATTCATACGAAGCATG |
